# Association of cyclophilins and cardiovascular risk factors in coronary artery disease

**DOI:** 10.3389/fphys.2023.1127468

**Published:** 2023-03-01

**Authors:** Sandra Gegunde, Amparo Alfonso, Rebeca Alvariño, Nadia Pérez-Fuentes, Jeremías Bayón-Lorenzo, Eva Alonso, Raymundo Ocaranza-Sánchez, Rosa Alba Abellás-Sequeiros, Melisa Santás-Álvarez, Mercedes R. Vieytes, Carlos Juanatey-González, Luis M. Botana

**Affiliations:** ^1^ Departamento de Farmacología, Facultad de Veterinaria, Universidad de Santiago de Compostela, Lugo, Spain; ^2^ Grupo Investigación Biodiscovery, IDIS, Lugo, Spain; ^3^ Servicio de Cardiología, Hospital Universitario Lucus Augusti, Lugo, Spain; ^4^ Fundación Instituto de Investigación Sanitaria de Santiago de Compostela (FIDIS), Lugo, Spain; ^5^ Departamento de Fisiología, Facultad de Veterinaria, Universidad de Santiago de Compostela, Lugo, Spain

**Keywords:** cyclophilins, cardiovascular disease, inflammation, cardiovascular risk factors, type 2 diabetes, dyslipidemia

## Abstract

Cyclophilins are chaperone proteins that play important roles in signal transduction. Among them, cyclophilins A, B, C, and D were widely associated with inflammation and cardiovascular diseases. Cyclophilins A and C have been proposed as coronary artery disease biomarkers. However, less is known about their relationship with cardiovascular risk factors. Therefore, this study aimed to determine the association between cyclophilin A, B, C, and D and cardiovascular risk factors in coronary artery disease. Serum levels of cyclophilins were measured in 167 subjects (subdivided according to cardiovascular risk factors presence). This study reveals that cyclophilin A and C are elevated in patients regardless of the risk factors presence. Moreover, cyclophilin B is elevated in male patients with hypertension, type 2 diabetes, or high glucose levels. In addition, cyclophilins A, B, and C were significantly correlated with cardiovascular risk factors, but only cyclophilin B was associated with type 2 diabetes. The multivariate analysis strengthens the predictive value for coronary artery disease presence of cyclophilin A (>8.2 ng/mL) and cyclophilin C (>17.5 pg/mL) along with the cardiovascular risk factors tobacco, hypertension, dyslipidemia, and high glucose and cholesterol levels. Moreover, the risk of coronary artery disease is increased in presence of cyclophilin B levels above 63.26 pg/mL and with hypertension or dyslipidemia in male patients. Consequently, cyclophilins A and C serum levels are reinforced as useful coronary artery disease biomarkers, meanwhile, cyclophilin B is a valuable biomarker in the male population when patients are also suffering from hypertension or dyslipidemia.

## 1 Introduction

Coronary artery disease (CAD) is one of the main causes of death in the world, despite the latest advances in prevention, diagnosis, and treatment (Zhou et al., 2016). CAD is an atherosclerotic cardiovascular disease (CVD) with an inflammatory component. In this sense, atherosclerosis is a chronic inflammatory disorder of the arteries promoted by lipids. Atherosclerotic plaques are mainly located in the intima of medium and large arteries. During the atherosclerosis plaque development, endothelial cells, lymphocytes, smooth muscle cells, and macrophages are involved, from the early formation of foam cells to the development and consolidation of plaques (Wolf and Ley, 2019). Moreover, modifications of low-density lipoprotein (LDL), such as oxidation or desialylation, enhance the intracellular accumulation of cholesterol or triglycerides and are implicated in the inflammatory process (Aguilar Diaz de Leon et al., 2021). When the atherosclerotic plaque grows up the lumen of arteries decreases, reducing the blood flow (Bezsonov et al., 2021). This pathology is a continuous process throughout human life, it normally begins at an early age, but it is not until years later that clinical symptoms begin to appear (Fulop et al., 2018). This progressive disorder is clinically manifested by stable or unstable angina, myocardial infarction (MI), or sudden myocardial death (Malakar et al., 2019). Hypertension (HTA), diabetes, cigarette smoking, hyperglycemia, and lipid abnormalities are the main modifiable risk factors for CAD (Wolf and Ley, 2019; Mone et al., 2021; Cui et al., 2022). Diabetes, especially type 2 diabetes (T2D), is the major risk factor involving CAD. Moreover, 75% of patients with T2D die due to CVD complications, including CAD (Naito and Miyauchi, 2017). Diabetes is a metabolic chronic disease with an inflammatory component in response to high blood glucose levels (McKinley, 2019). HTA is a common comorbidity in patients with diabetes. The association of T2D, hyperglycemia, and HTA increases the possibility of cardiovascular events, being together a high cardiovascular risk. Therefore, the early diagnosis of CAD and its risk factors is critical for enhancing both prevention and treatment, especially in patients with T2D or HTA (Thomopoulos et al., 2014). In this regard, high-sensitivity C-reactive protein (hs-CRP), d-dimer, fibrinogen, and brain atrial natriuretic peptide are used as predictors of CVD events (Ndrepepa et al., 2013; Gul et al., 2016). Nevertheless, these biomarkers are not specific, and they are generally elevated in inflammatory diseases (Wang et al., 2006).

In this sense, the search for new specific biomarkers has led to the study of cyclophilins (Cyps). This family of proteins, also known as immunophilins, has peptidyl-prolyl *cis/trans* isomerase (PPIase). These proteins act as molecular chaperones and are involved in cell signaling and protein folding and trafficking. High levels of extracellular cyclophilins have been found in several inflammatory-based diseases, such as rheumatoid arthritis or sepsis. Moreover, among them, CypA, B, C, and D have been associated with atherosclerosis and CVD (Aumuller et al., 2010; Kumari et al., 2013; Perrucci et al., 2015). Both CypA and B are secreted upon inflammatory stimulus and oxidative stress by several cells (Satoh et al., 2010). Extracellularly, these proteins have a pathological role in human CVD. They act as cytokines and have chemoattractant activity for immune cells (Perrucci et al., 2015). CypA stimulates the adhesion, migration, and differentiation of monocytes participating in atherosclerosis progression (Ramachandran et al., 2016). Moreover, CypA mediates inflammation, ROS generation, and matrix degradation in CVD by binding to the membrane receptor CD147 (Sanchez et al., 2016; Chen et al., 2018). CypA has been described as a biomarker with a high diagnosis and prognosis value for CAD (Satoh et al., 2013; Ohtsuki et al., 2017). Furthermore, CypA levels are correlated with the severity of the disease (Xue et al., 2018). Extracellular CypB, in addition to producing immune cell chemotaxis, also induces their adhesion to damaged tissues, aggravating the inflammatory process. High CypB serum levels have been found in patients with rheumatoid arthritis and metabolic syndrome (Zhang et al., 2017). However, despite the relationship of this protein with inflammatory processes, no changes in CypB levels were found in patients with CAD (Alfonso et al., 2019; Bayon et al., 2020). Fewer is known about CypC. Nevertheless, our group has identified high CypC levels in the serum of both acute and chronic CAD patients (Alfonso et al., 2019; Bayon et al., 2020). Furthermore, after 12 months, CypC serum levels remain elevated in these patients (Bayon et al., 2021). In addition, CypC is upregulated in a rat model of brain ischemia (Shimizu et al., 2005). Although the extracellular functions of this protein are unknown, there is a clear correlation between CypC and CVD. Besides, high intracellular levels of CypA, B, and C in human T-cells were recently associated with CAD (Gegunde et al., 2021). CypD has a key role in chronic inflammation and CVD. This protein is the main regulator of the mitochondrial permeability transition pore (mPTP) opening and the mitochondrial calcium homeostasis. However, CypD has been implicated in the pathogenesis of neurodegenerative disorders, muscular dystrophy, and ischemia-reperfusion (IR) injury in the heart, brain, and kidney (Galber et al., 2020). Moreover, mitochondrial DNA mutations are implicated in several diseases, including atherosclerosis (Bezsonov et al., 2021). Nevertheless, likewise CypB, serum CypD levels remain unchanged in CAD patients (Alfonso et al., 2019; Bayon et al., 2020).

The key step in preventing major cardiovascular events is to keep the cardiovascular risk factors under control. Therefore, knowing the implications of CypA, B, C, and D in CVD, this study aimed to deeply investigate their relationship with cardiovascular risk factors in CAD patients, to better understand the disease and predict major events.

## 2 Methods

### 2.1 Population study

**FIGURE 1:**
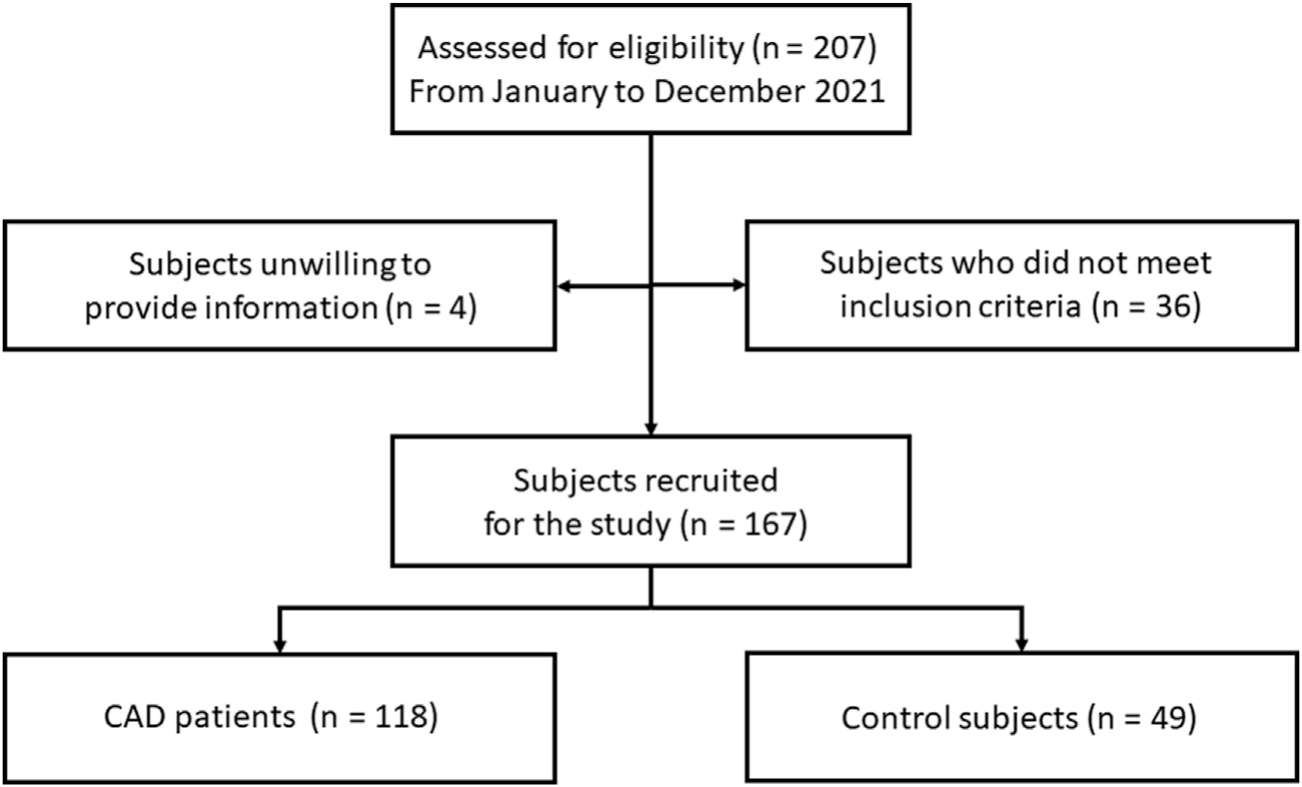
Flow chart of the study. CAD: coronary artery disease.

An observational study about serum levels of Cyps and their association with cardiovascular risk factors was conducted in patients with CAD. CAD patients were referred from the Cardiology Department of Hospital Universitario Lucus Augusti in Lugo, Spain, from January 2021 to December 2021 (Figure 1). A total of 118 patients with CAD were included in the study. CAD disease was defined as prior MI, coronary revascularization, or angiographic documentation of any significant coronary artery stenosis. Coronary angiograms were evaluated by experienced cardiologists, who were blinded to the patient data. A narrowing of the artery lumen by more than 51% of the diameter was considered clinically significant for CAD. Critically or hemodynamically unstable patients, patients with valvular disease, or congestive heart failure were excluded. Also, 49 control volunteers were included in the study. Control volunteers were subjects without known atherosclerosis disease, with normal angiography findings, normal serum cardiac biomarkers, and without cardiovascular risk factors. Participants with chronic or acute inflammatory diseases, cancer, autoimmune diseases, and rheumatic disease were excluded. Initially, it was 65 subjects in the control group, but only 49 met the inclusion criteria. All participants belong to the public healthcare area of Lugo, Spain. The population to be studied was designed according to CAD prevalence in this area. The institutional and regional ethical board approved the study according to the principles outlined in the Declaration of Helsinki. Voluntary written informed consent was obtained from the participants in the present study.

### 2.2 Baseline measurements

Information on general vital status, significant clinical data, and medical history were obtained from all participants. Also, they respond to the personal questionnaire. HTA, T2D, dyslipidemia (DL), tobacco, age, and sex (male) were assessed as cardiovascular risk factors. The lipid-lowering, antihypertensive, hypoglycemic, and antiaggregant or antiplatelet drug consumption was considered. Hypertension was considered when blood pressure was ≥ 140/90 mmHg. Diabetes was considered when fasting glucose levels were ≥ 126 mg/dL or if hypoglycemic treatment or insulin was used. DL was considered if low-density lipoprotein cholesterol (LDLc) levels were ≥ 140 mg/dL or high-density lipoprotein cholesterol (HDLc) levels were ≤ 40 mg/dL, or if an antilipidemic treatment was used. Biochemical parameters (levels of glucose, HDLc, and LDLc) were measured in the Analysis Service from Lucus Augusti Hospital using an ADVIA Clinical Chemistry System (Siemens Healthcare).

### 2.3 Blood sampling protocol

Peripheral blood samples were obtained from each patient as before described (Alfonso et al., 2019). Briefly, Blood samples were allowed to clot for 20 min at room temperature before being centrifuged at 3,000 rpm for 10 min at 4 °C. Serum supernatant fractions are collected and stored at −80°C until needed for Cyps level analysis. Samples were thawed once.

Measurement of Cyclophilin A, B, C, and D serum levels.

Levels of serum CypA (Human Cyclophilin A ELISA kit; CSB-E09920H; Cusabio), CypB (Human Cyclophilin B ELISA kit; CSB-E11218H: Cusabio), CypC (Human Cyclophilin C ELISA kit; CSB-EL018473HU; Cusabio) and CypD (Human Cyclophilin D ELISA kit; E-EL-H1936; Elabscience) were measured using ELISA regarding manufacturer’s instructions and as described before (Alfonso et al., 2019). Before carrying out the ELISA experiments for the measurement of Cyp levels, serum samples were brought to room temperature, vortexed, and centrifuged. Absorbance measurements were done in a microplate reader at 450 and 540 nm for CypA, B, and C, and 450 nm in the case of CypD). The range of determination was 3.12–200 ng/mL for CypA; 31.25–2,000 pg/mL for CypB; 23.5–1,500 pg/mL for CypC and 62.5–4,000 pg/mL for CypD. Serum levels below the lower limit of determination were undetectable and were considered as 0 pg/mL for statistical analysis. The intra and inter-assay coefficients of variation of the ELISA kits were < 10%. No cross-reactivity was observed between Cyp antibodies.

### 2.4 Statistical analysis

Participants were divided according to cardiovascular risk presence. Summary statistics were generated and presented as percentages for categorical variables and mean ± SEM for continuous variables. Normality was evaluated using the Kolmogorov-Smirnov test (with Lilliefors correction). Continuous variables with normal distribution were compared between groups using a *t*-test (including Levene´s test to assess the equality of variance). Non-parametric variables were compared using the Mann-Whitney test. Categorical variables were compared using the Pearson χ2 test. To compare the differences between the groups, ANOVA or the Kruskal-Wallis (in the case of non-parametric variables) test was used, followed by *post hoc* tests. Correlations between Cyps levels and cardiovascular risk factors were analyzed using bivariate analysis with Spearman rank correlation coefficient in the case of categorical variables (sex, age >50 years, active smoker, DL, HTA, T2D, glucose >100 mg/dL, TG > 200 mg/dL, total cholesterol >200 mg/dL, LDLc >100 mg/dL, and HDLc <50 mg/dL) and the principal component analysis was used for calculating the correlations between numeral variables (laboratory parameters of glucose, triglycerides; TG, total cholesterol, LDLc, and HDLc). After a significant crude correlation was found, multiple linear regression models with a subsequent backward stepwise were used to assess the association of CypA, CypB, CypC, and/or CypD serum levels with cardiovascular risk factors. All stepwise selection models used a *p* < 0.05 level for entry and a *p* < 0.10 for removal. The variables included in these models were based on the previous simple regression models. The association was measured by the odds ratio (OR) and their 95% confidence interval (CI). To obtain the cut-off points of CypB levels for logistic regression, receiver-operating-characteristic curves (ROC) were used. Statistical analyses were performed using SPSS software version 25.0 (IBM Corp, NY, United States). *p* ≤ 0.05 was considered statistically significant.

## 3 Results

**FIGURE 2:**
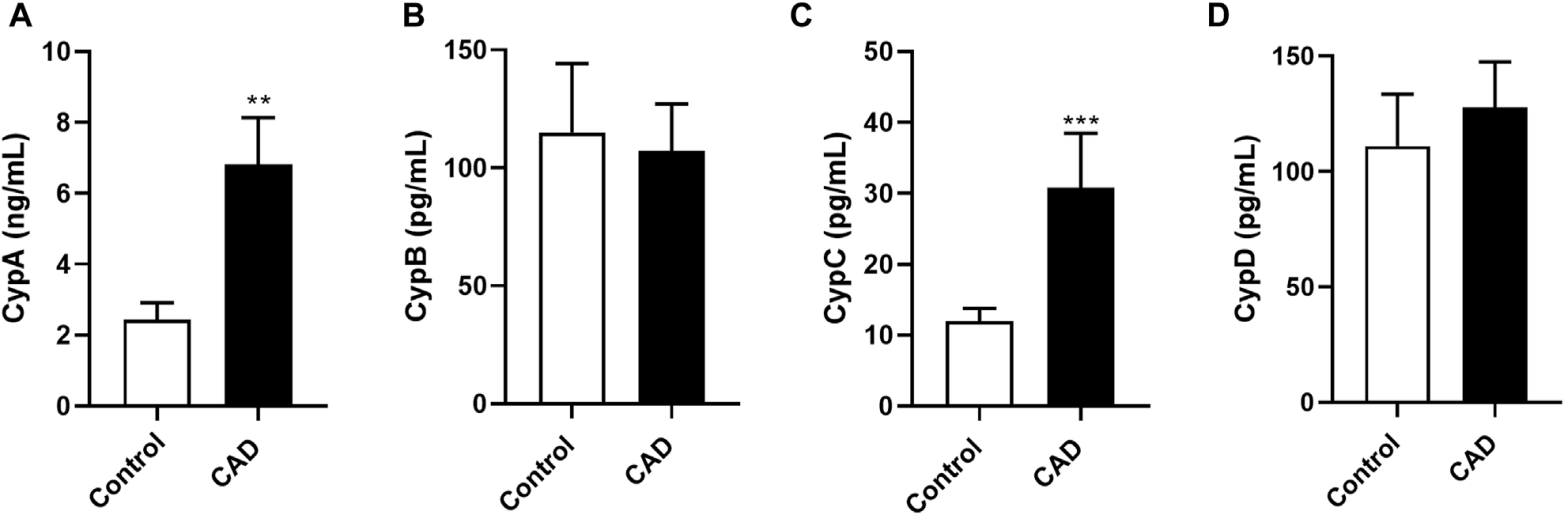
Cyclophilin A, B, C, and D serum levels in control subjects and patients with coronary artery disease with no associated risk factor. Serum levels of CypA **(A)**, CypB **(B)**, CypC **(C)** and CypD **(D)** in control subjects (n = 49) and CAD patients (n = 16) with any associated risk factor. Data are shown as mean ± SD. The values are shown as the difference between control subjects versus CAD patients, **p < 0.01 and ***p < 0.001 using the Mann-Whitney test. CAD: coronary artery disease; CypA; cyclophilin A; CypB: cyclophilin B; CypC: cyclophilin C; CypD: cyclophilin **(D)**.

A total of 167 subjects were enrolled in the present study, as Figure 1 shows. Among all, 118 were diagnosed with CAD (82% men) and 49 belonged to the control group (53% men). The demographic, clinical, and biochemical data of the participants were collected and the most relevant data for this study were summarized in table 1. Then, the CAD population was divided according to the presence of cardiovascular risk factors sex (male), tobacco, HTA, DL, and T2D. Among the patients diagnosed with CAD, 33 are active smokers, 64 have HTA, 85 have DL, and 27 are suffering from T2D. Therefore, CypA, B, C, and D levels were first measured in the population with no associated risk factors (Figure 2). In this population, CypA serum levels were significantly high in CAD patients (6.80 ± 1.6 ng/mL) compared with controls (2.42 ± 0.48 ng/mL; *p* < 0.001). In the case of serum CypB levels, no statistical differences were observed when controls (114.91 ± 29.32 pg/mL) were compared with the CAD group (107.07 ± 19.94 pg/mL). Like CypA, CypC was a threefold increase in CAD subjects compared to control subjects (*p* < 0.001). Finally, no significant changes in CypD levels of control subjects *versus* CAD patients were observed.

**TABLE 1:** demographic, and biochemical characteristics.

	Control subjects (n = 49)	CAD patients (n = 118)	
**Sex (male) (%)**	53.06	82.2	
**Age (years)**	56.3 ± 1.45	63.69 ± 1.15	
**Laboratory parameters**			** *p*-value**
**Total cholesterol (mg/dL)**	201.14 (±4.56)	171.35 (±5.09)	<0.001
**LDLc (mg/dL)**	120.77 (±4.13)	103.44 (±4.30)	<0.001
**HDLc (mg/dL)**	57.77 (±2.58)	37.99 (±1.11)	<0.001
**TG (mg/dL)**	107.17 (±6.78)	154.81 (±8.39)	<0.001
**Glucose (mg/gL)**	90.54 (±5.48)	125.47 (±5.20)	<0.001

Data are shown as average ± SEM, or as a percentage. CAD: coronary artery disease; LDLc: low-density lipoprotein cholesterol; HDLc: high-density lipoprotein cholesterol; TG: triglycerides. Student’s t-test or Mann-Whitney test were used to perform statistical analysis. Categorical variables were compared using Χ^2^ test. Significant differences: *p* < 0.05.

**FIGURE 3:**
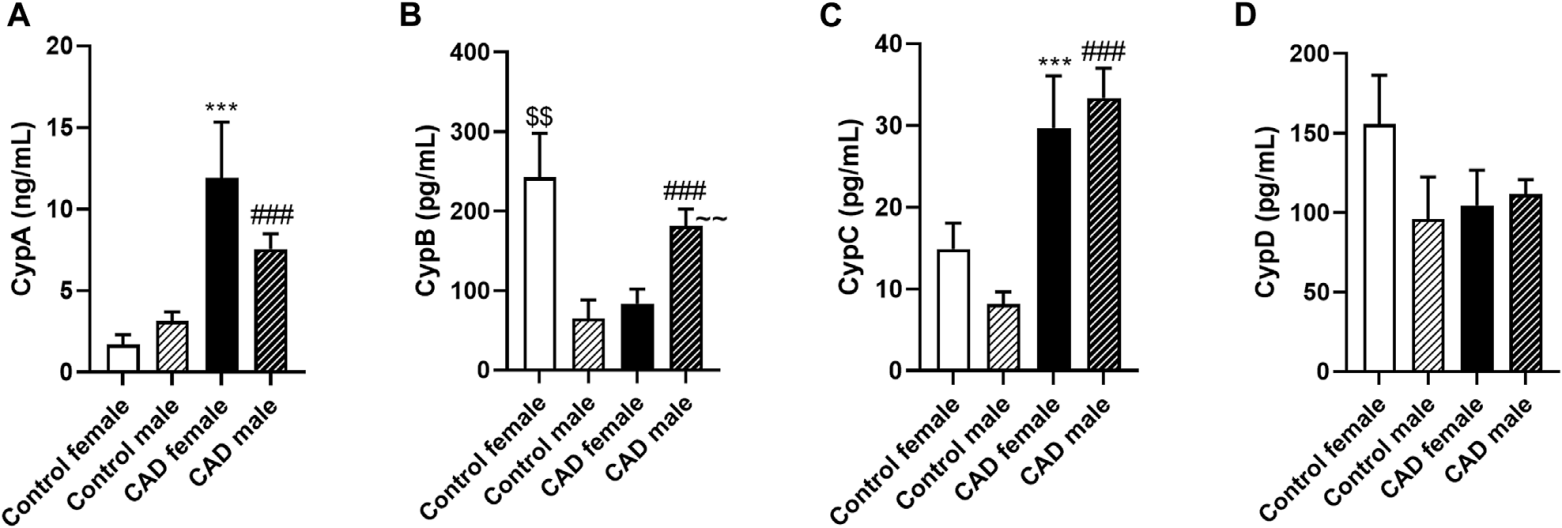
Cyclophilin A, B, C, and D serum levels in control subjects and patients with coronary artery disease subdivided according to sex. Serum levels of CypA **(A)**, CypB **(B)**, CypC **(C)** and CypD **(D)** in control female subjects (n = 23), control male (n = 26), male CAD patients (n = 97) female CAD patients (n = 21). Data are shown as mean ± SD. The values are shown as the difference between control female subjects versus female CAD patients, ***p < 0.001, or bewteen control male subjects versus male CAD patients, ###*p* < 0.001, or between control female versus control male, $$ *p* < 0.01, or between female CAD patients versus male CAD patients, ∼∼ *p* < 0.01 using the Mann-Whitney test. CAD: coronary artery disease; CypA; cyclophilin A; CypB: cyclophilin B; CypC: cyclophilin C; CypD: cyclophilin **(D)**.

Therefore, the next step was to evaluate Cyps levels according to cardiovascular risk factors (sex, tobacco, DL, HTA, and T2D) presence. When the total population was divided according to sex, there were no differences in CypA or CypC levels between control women and control men (Figures 3A, C). Furthermore, there were no statistical differences between the serum levels of CypA or CypC in women and men with CAD. Nevertheless, both CypA and CypC were increased in women with CAD when compared with control women (Figures 3A, C, *p* < 0.001). Moreover, CypA and CypC levels were higher in men with CAD than in male controls (Figures 3A, C, *p* < 0.001). CypB levels increased 4-fold in control women compared to control men (Figure 3B, *p* < 0.01). However, CypB levels were higher in men with CAD compared to women with CAD or men without CAD (*p* < 0.001). Serum levels of CypD remain unchanged in all groups.

There is a discrepancy in serum CypB levels between males and females. These differences between sex could be due to female hormones. In this sense, CypB has been associated with gene expression of hormone receptors in women (Fang et al., 2009). Consequently, to avoid interferences, only the male population was used in subsequent studies for CypB. Therefore, the serum CypB levels were re-evaluated in the male population with no associated risk factors. In this population, CypB serum levels were significantly elevated in CAD patients (194.31 ± 25.52 pg/mL) compared with controls (64.24 ± 23.06 pg/mL; *p* < 0.001; data not shown).

**FIGURE 4:**
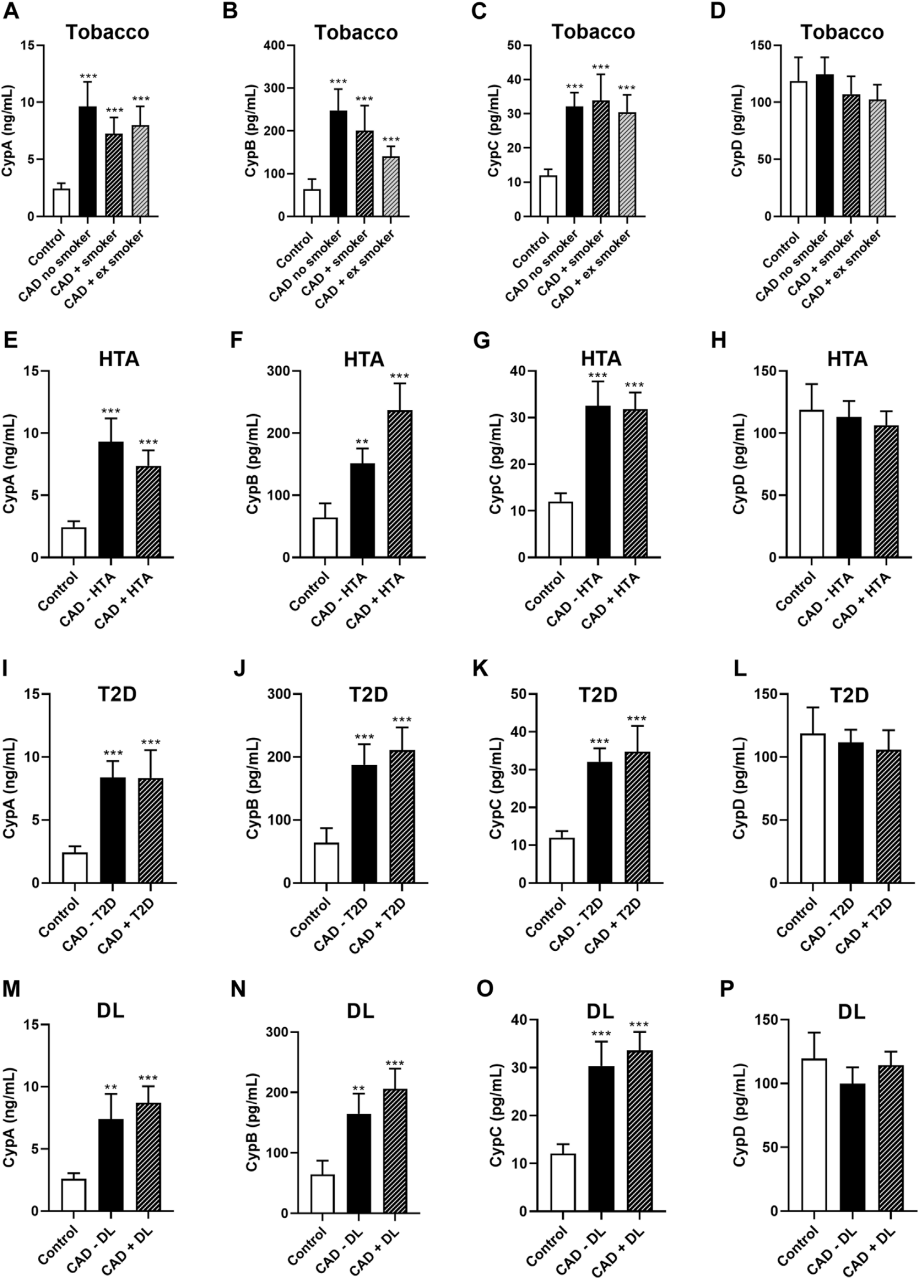
Cyclophilin A, B, C, and D serum levels in control subjects and patients with coronary artery disease subdivided according to smoking status, HTA, T2D, or DL. Serum levels of CypA **(A)**, CypB (in male population) **(B)**, CypC **(C)**, and CypD **(D)** in controls (n = 49, *n = 26), CAD patients no smokers (n = 50, *n = 33), smoker CAD patients (n = 29, *n = 27) and ex-smoker CAD patients (n = 39, n = 37). Serum levels of CypA **(E)**, CypB (in male population) **(F)**, CypC **(G)**, and CypD **(H)** in controls (n = 49, *n = 26), CAD patients without HTA (n = 54, *n = 46), and CAD patients with HTA (n = 64, *n = 51). Serum levels of CypA **(I)**, CypB (in male population) **(J)**, CypC **(K)**, and CypD **(L)** in controls (n = 49, *n = 26), CAD patients without T2D (n = 91, *n = 73), and CAD patients with T2D (n = 27, n = 24). Serum levels of CypA **(N)**, CypB (in male population) **(M)**, CypC **(O)**, and CypD **(P)** in controls (n = 49, *n = 26), CAD patients without DL (n = 85, *n = 72), and CAD patients with DL (n = 33, *n = 25). ANOVA or the Kruskal-Wallis (in the case of non-parametric variables) test was used, followed by *post hoc* tests. *Studies of CypB in the male population. CAD: coronary artery disease; CypA; cyclophilin A; CypB: cyclophilin B; CypC: cyclophilin C; CypD: cyclophilin D; DL: dyslipidemia; HTA: hypertension: T2D: type 2 diabetes.

Then, CAD patients were divided based on smoking status (no smoker, active smoker, and ex-smoker; Figure 4). Although CypA and CypC levels were increased in CAD patients compared to controls (Figures 4A, C, *p* < 0.001), no differences were found between CAD groups according to smoking status. In the male population, CypB was significantly elevated in CAD patients compared to controls (Figure 4B, *p* < 0.001). However, no differences in CypB levels were found among CAD patients based on smoking status. Moreover, no significant variances in CypD serum levels between CAD groups or between control and CAD groups were observed (Figure 4D). Afterward, HTA was considered. In this study population, CypA and C levels were augmented in the serum of all CAD patients compared to controls, regardless of the presence of this risk factor (Figures 4E, G). Moreover, CypB levels were also augmented (in the male population) in CAD patients with or without HTA *versus* controls (Figure 4F; *p* < 0.01). Despite the CypB serum levels were increased in CAD patients with HTA than in CAD patients without HTA, this difference was not statistically significant. Nevertheless, no major differences were found regarding CypD levels in this population (Figure 4H). Next, Cyps levels were measured in CAD patients in the presence or absence of the cardiovascular risk factor T2D. Both CypA and CypC were constant in CAD patients regardless of the presence of T2D, meanwhile, these levels were statistically increased in both CAD groups compared to control levels (Figures 4I, K; *p* < 0.001). CypB was also augmented in the male population with CAD plus T2D and CAD patients without T2D compared with the control group (Figure 4J; *p* < 0.001). Nevertheless, CypD serum levels were unaltered in all groups (Figure 4L). Finally, Cyps levels were measured according to DL status in CAD patients. CypA and CypC levels were statistically elevated in CAD patients with or without DL compared to controls (Figures 4m and 4o, *p* < 0.01). CypB was also increased in male CAD patients regardless of DL status (Figure 4n; *p* < 0.01). Meanwhile, there was no change in CypD levels in DL patients relative to non-DL patients or controls (Figure 4p). Therefore, CypA and C were elevated in general in CAD patients regardless of the presence of cardiovascular risk factors. Meanwhile, CypB was raised in female controls, while in the CAD group, it was increased in men.

Subsequently, the correlation between Cyps levels and cardiovascular risk factors was calculated. Bivariate correlation analysis was used to assess the correlation between Cyps and categorical variables (sex–male-, age >50 years, active smoker, HTA, DL, T2D, glucose levels >200 mg/dL, total cholesterol levels >200 mg/dL, LDLc levels >100 mg/dL and HDLc levels <50 mg/mL). The cut-off of biochemical parameters was chosen following the recommendations of the European guidelines for CVD prevention (Visseren et al., 2021). Bivariate correlation analysis shows, in table 2, a positive correlation between CypA and CypC, sex, age >50 years, active smoker, DL, and glucose >100 mg/dL (*p* < 0.007). CypB was associated, in the male population, with age >50 years, HTA, DL, T2D and glucose >100 mg/dL. Moreover, CypC was positively associated with CypA, age >50 years, active smoker, HTA, DL, and glucose >100 mg/dL (*p* < 0.05). Nevertheless, CypD was only correlated with total cholesterol >200 mg/dL.

**TABLE 2:** Correlations between cyclophilins and cardiovascular risk factors.

Correlations	CypA	CypB*	CypC	CypD
**CypA**	1	-	0.326 (*p* < 0.001)	
**CypB**	-	1	-	-
**CypC**	0.326 (*p* < 0.001)	-	1	
**CypD**		-		1
**Sex (male)**	0.251 (*p* = 0.002)	-		
**Age (> 50 years)**	0.381 (*p* < 0.001)	0.304 (*p* < 0.001)	0.274 (*p* < 0.001)	
**Active smoker**	0.220 (*p* = 0.006)		0.172 (*p* = 0.030)	
**HTA**		0.304 (*p* < 0.001)	0.255 (*p* = 0.001)	
**DL**	0.248 (*p* = 0.002)	0.199 (*p* = 0.016)	0.257 (*p* = 0.001)	
**T2D**		0.203 (*p* = 0.036)		
**Glucose (> 100 mg/dL)**	0.216 (*p* = 0.007)	0.249 (*p* = 0.009)	0.347 (*p* < 0.001)	
**Total Cholesterol (> 200)**				0.178 (*p* = 0.025)
**LDLc (> 100 mg/dL)**				
**HDLc (< 50 mg/dL)**				

Spearman’s correlation coefficient. DL: dyslipidemia. T2D: type 2 diabetes; LDLc: low-density lipoprotein cholesterol; HDLc: high-density lipoprotein cholesterol; HTA: hypertension; TG: triglycerides. Significant differences *p* < 0.05. N = 167. n = 167. *n = 123.

**FIGURE 5:**
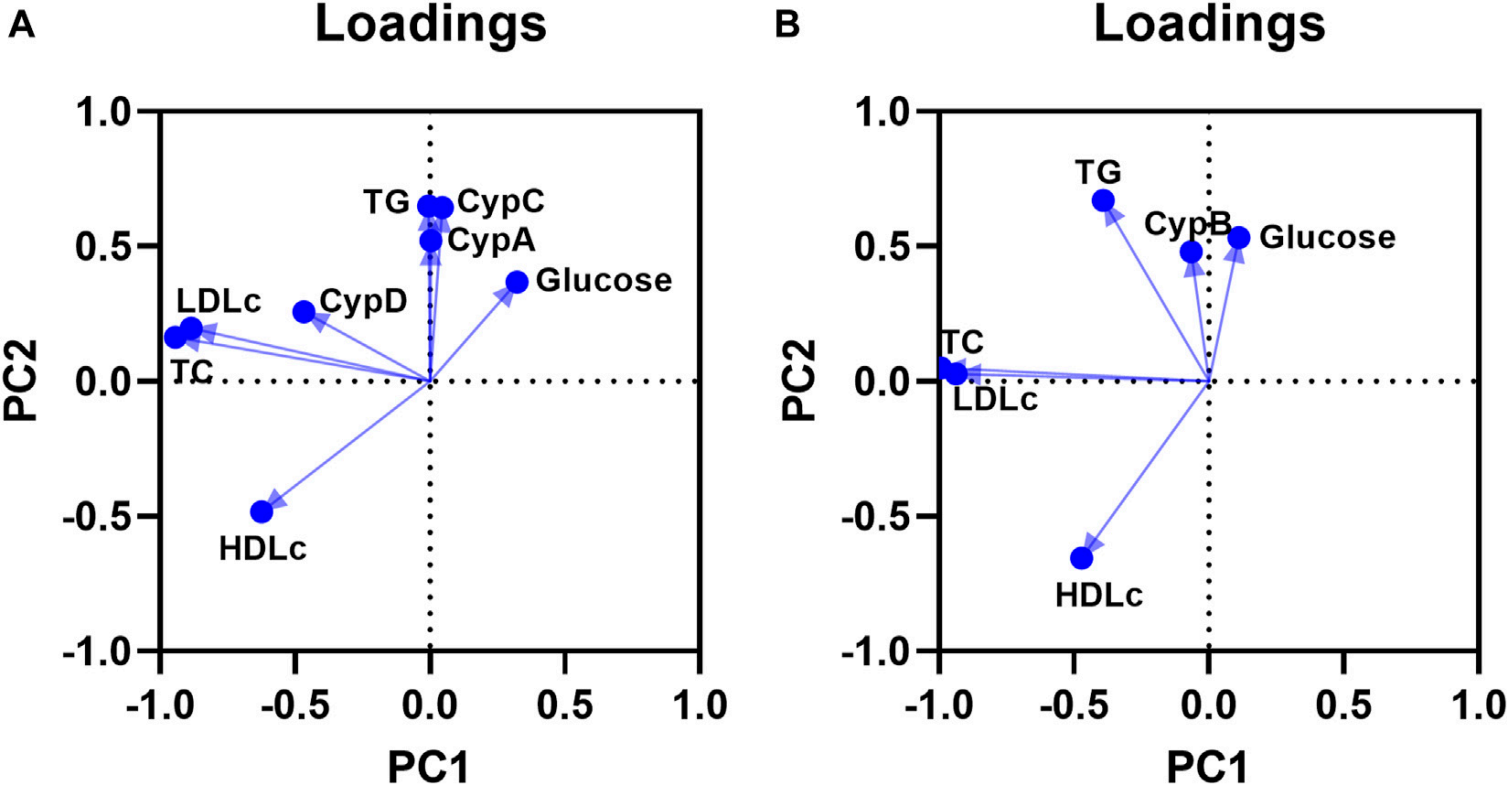
Principal component analysis (PCA) plot for CypA, C and D, and laboratory parameters **(A)**. Grouping of the variables in two principal components. PCA was performed using TG, total cholesterol, LDLc, HDLc, glucose, CypA, C, and D serum levels. The two PC are shown, and the percentage of variation accounted for PC1 is 29.87% and for PC2 is 20.09%. n = 167. PCA plot for CypB and laboratory parameters in male population **(B):** PCA was performed using TG, total cholesterol, LDLc, HDLc, glucose and CypB serum levels. The two PC are shown, and the percentage of variation accounted for PC1 is 37.65% and for PC2 is 23.25%. n = 123. CypA: cyclophilin A; CypB: cyclophilin B; CypC: cyclophilin **(C)** CypD: cyclophilin D; LDLc: low-density lipoprotein cholesterol; HDLc: high-density lipoprotein cholesterol; TC: total cholesterol; TG: triglycerides.

Then, the principal component analysis (PCA) was carried out to identify the grouping patterns and correlations among the laboratory parameters (glucose, TG, total cholesterol, LDLc, and HDLc) and CypA, C, and D (Figure 5A). Two principal components (PC) with eigenvalues >1 explained 49.96% of the total variances. PC 1 represents the variation for total cholesterol and LDLc levels and PC2 for TG and CypC. The ordination showed pronounced differentiation between total cholesterol and LDLc with glucose, TG and CypA, and CypC. CypA and C were closer to TG. Moreover, HDLc was negatively correlated with glucose, TG, CypA, and CypC. Therefore, CypA and C are associated with glucose and TG serum levels and negatively associated with HDLc levels. Then the PCA was done in the male population to assess the correlations between CypB and the laboratory parameters (Figure 5B). Two PC represent a 60.90% variation of the dataset (eigenvalues >1). PC 1 represents the variation for Total Cholesterol and LDLc, meanwhile PC 2 for TG and HDLc. The PCA showed differentiation between TG, CypB, and glucose with total cholesterol and LDLc. Furthermore, CypB was close to TG and glucose and negatively correlated with HDLc. In this sense, CypB was associated with TG and glucose and negatively associated with HDLc.

Given these results, the next step was to study the relationship between risk factors and Cyps in the presence of CAD. For this purpose, the cut-off points of Cyps for CAD presence were used. Cut-off points for CypA >8.2 ng/mL and C > 17.5 pg/mL were obtained from ROC curves of our preliminary study and validated in the present study in CAD patients with or without cardiovascular risk factors associated (Alfonso et al., 2019). The cut-off for CypA > 8.2 ng/mL had a specificity of 95.5% and a sensitivity of 32.5% with a positive predictive value (PPV) of 86.7% and a negative predictive value (NPV) of 60.9%. The cut-off point for CypC >17.5 pg/mL provided a specificity of 88.6% and a sensitivity of 70% with a PPV of 84.8% and NPV of 76.5% (Alfonso et al., 2019). In the case of CypB, a cut-off point >63.26 pg/mL was calculated in the present study in the male population using ROC curves (data not shown). This cut-off point has a specificity of 78.3% and a sensitivity of 66.7% with a PPV of 91.8% and NPV of 60.86%. Meanwhile, from the ROC curve analysis, the cut-off point for CypD could not be obtained, because it does not significantly predict the presence of CAD. Therefore, CypD was not used in the following analyses.

Then, the cardiovascular risk factors and Cyps were combined in a single-logistic regression model. So, using the presence or absence of CAD as a state variable, univariate analyses were performed. Then, to check if Cyps cut-off points could predict the presence of CAD, a univariate analysis was done (Table 3). CypA >8.2 ng/mL were correlated with the presence of CAD with an OR of 3.58 (*p* = 0.024). Moreover, the risk of suffering from CAD was increased more than 9 times (*p* < 0.001) when the levels of CypC were >17.5 pg/mL. In the univariate analysis, the tobacco, HTA, DL, and high glucose levels were also correlated with the presence of CAD (with OR > 7; *p* < 0.001). As table 3 (analysis 1) shows when the multivariate analysis was performed with the risk factors tobacco, HTA, DL, and the biochemical parameters glucose (cut-off point >100 mg/dL) and total cholesterol (cut-off point >200 mg/dL) with CypA, this Cyp misses its correlation with the presence of CAD, meanwhile the risk factors (active smoker, HTA, DL, and glucose >100 mg/dL) were significantly associated with CAD (*p* < 0.027). Moreover, total cholesterol >200 mg/dL was negatively associated with the disease. When CypC was included, instead of CypA (table 3, analysis 2), a strong association of CypC >17.5 pg/mL with the presence of CAD (OR = 16.51; *p* = 0.002) together with the risk factors was observed. Furthermore, when CypA and CypC were combined with these risk factors the OR value for CypC was increased (Table 3, analysis three; OR = 18.02; *p* < 0.001) and was significantly associated with the presence of CAD and risk factors. The association between the active smoker, HTA, DL, and CAD when CypA and CypC are present in the multivariate analysis was in line with previous results (Alfonso et al., 2019). However, it is the first time that these Cyps were significantly correlated with serum levels of total cholesterol (>200 mg/dL) or glucose (>100 mg/dL) and CAD presence.

**TABLE 3:** Logistic regression analysis for the association of risk factors and cyclophilin A and C with CAD.

	Univariate analysis		Multivariate analysis (Zhou et al., 2016)		Multivariate analysis (Wolf and Ley, 2019)		Multivariate analysis (Aguilar Diaz de Leon et al., 2021)	
**Variable**	OR [95% CI]	*p*-value	OR [95% CI]	*p*-value	OR [95% CI]	*p*-value	OR [95% CI]	*p*-value
**Active smoker**	7.47 [3.31–16.86]	<0.001	36.03 [4.75–272.79]	0.001	39.54 [4.81–324.63]	0.001	53.22 [5.69–497.78]	<0.001
**HTA**	18.17 [5.35–61.72]	<0.001	5.98 [1.22–29.20]	0.027	7.08 [1.10–45.39]	0.039	7.40 [1.13–48.36]	0.037
**DL**	18.46 [7.18–47.44]	<0.001	20.57 [5.00–84.50]	<0.001	24.49 [4.73–126.78]	<0.001	19.35 [3.59–104.28]	0.001
**Glucose (> 100 mg/dL)**	35.15 [8.34-156-64]	<0.001	86.79 [11.99–627.94]	<0.001	50.45 [6.72–378.55]	<0.001	57.85 [6.81–490.90]	<0.001
**Total Cholesterol (> 200)**	0.30 [0.14–0.65]	0.002	0.13 [0.02–0.72]	0.020	0.08 [0.01–0.596]	0.013	0.06 [0.00–0.50]	0.010
**CypA (> 8.2 ng/mL)**	3.58 [1.18–10.87]	0.024	4.54 [0.496–41.70]	0.180			5.70 [0.53–60.51]	0.148
CypC (>17.5 ng/mL)	9.57 [4.07–22,49]	<0.001			16.51 [2.90–93.80]	0.002	18.02 [3.06–105.89]	0.001

Univariate and multivariate analysis. Multivariate analysis one included: Active smoker, HTA, DL, glucose >100 mg/dL, total cholesterol >200 mg/dL, and CypA >8.2 ng/mL; Cox and Snell *R* ^2^: 0.541; Nagelkerke *R* ^2^: 0.775. Multivariate analysis two included: Active smoker, HTA, DL, glucose >100 mg/dL, total cholesterol >200 mg/dL, and CypC >17.4 pg/mL; Cox and Snell *R* ^2^: 0.576; Nagelkerke *R* ^2^: 0.821. Multivariate analysis three included: Active smoker, HTA, DL, glucose >100 mg/dL, total cholesterol >200 mg/dL, CypA >8.2 ng/mL, and CypC >17.5 pg/mL; Cox and Snell *R* ^2^: 0.584; Nagelkerke *R* ^2^: 0.830. CAD: coronary artery disease; CI: confidence interval; CypA: cyclophilin A; CypC: cyclophilin C; DL: dyslipidemia; HTA: hypertension; OD: odds ratio. Significant differences *p* < 0.05. n = 167.

Finally, CypB > 63.26 pg/mL showed a significant interaction when it was combined with tobacco, DL, and glucose (<100 mg/dL) with an OR of 9.10 (table 4, analysis one; *p* = 0.008). Also, these risk factors are significantly linked to CAD (*p* < 0.05). Moreover, the risk factor CypB continues to have prognostic value along with tobacco, HTA, and glucose (<100 mg/dL) (Table 4, analysis two; OR = 7.29; *p* = 0.011) for CAD. Therefore, the risk of CAD is increased in presence of CypA levels above 8.2 ng/mL or CypC above 17.5 pg/mL along with other cardiovascular risk factors. Furthermore, in the presence of CypB levels above 63.26 pg/mL and risk factors along with HTA or DL the risk of CAD is also elevated. Consequently, CypA and C serum levels are reinforced as useful CAD biomarkers in the general population. Meanwhile, CypB is a valuable biomarker of CAD when patients are also suffering from HTA or DL in males.

**TABLE 4:** Logistic regression analysis for the association of risk factors and cyclophilin B with CAD.

	Multivariate analysis (Zhou et al., 2016)		Multivariate analysis (Wolf and Ley, 2019)	
Variable	OR [95% CI]	*p*-value	OR [95% CI]	*p*-value
**Active smoker**	13.06 [2.32–73.50]	0.004	10.20 [2.12–49.05]	0.004
**HTA**			8.24 [1.29–52.45]	0.025
**DL**	8.10 [1.65–39.58]	0.010		
**Glucose (> 100 mg/dL)**	23.27 [2.67–202.56]	0.004	22.43 [2.43–206.74]	0.006
**CypB (> 63.26 ng/mL)**	9.10 [1.79–46.16]	0.008	7.29 [1.56–33.95]	0.011

Multivariate analysis one included: active smoker, DL, glucose >50 mg/dL, and CypB >63.26 pg/mL; Cox and Snell *R* ^2^: 0.454; Nagelkerke *R* ^2^: 0.701. Multivariate analysis two included: active smoker, HTA, glucose >50 mg/dL, and CypB >63.26 pg/mL. Cox and Snell *R* ^2^: 0.445; Nagelkerke *R* ^2^: 0.688. CAD: coronary artery disease; CI: confidence interval; CypB: cyclophilin B; DL: dyslipidemia; HDLc: high-density lipoprotein cholesterol; OD: odds ratio. Significant differences *p* < 0.05. n = 123.

## 4 Discussion

In the last 40 years, the number of CAD cases has been increasing. Nevertheless, despite advances in diagnosis and treatments, CAD is the leading cause of morbidity and mortality of CVD in western countries. This condition, which is associated with atherosclerosis, contributes to MI, heart failure, and sudden death. In addition to being an individual health burden, CVD is the costliest disease in Western Hemisphere (Slavin et al., 2021). Therefore, improving the means to identify patients at risk is necessary to enhance healthcare and maximize resource management. Circulating factors such as LDLc, hs-CRP, or atrial natriuretic peptide are established and used as serum biomarkers to evaluate the risk for adverse cardiac events. Nevertheless, as was previously mentioned, these circulating factors are frequently elevated in inflammation or in subjects without CVD (Wang et al., 2006). In this sense, in the present study, we evaluated the serum CypA, B, C, and D levels in CAD patients and their association with cardiovascular risk factors.

In the last years, Cyps were associated with CVD. In this sense, CypA mediates the progression of atherosclerosis by inducing the formation of foam cells and promoting adhesion, migration, and differentiation of monocytes, and activating endothelial cells (Ramachandran et al., 2016). Moreover, this protein mediates inflammation and contributes to the damage in IR and myocardial remodeling processes (Seizer et al., 2014). In the present study, high levels of CypA were found in the serum of patients with CAD. Furthermore, as was expected this protein was positively associated in CAD patients independently of the presence of cardiovascular risk factors. These data are consistent with previous studies, including data from our group (Satoh et al., 2013; Ramachandran et al., 2014; Bayon et al., 2020). In this sense, high levels of CypA provide prognostic information on the severity of CAD (Satoh et al., 2013; Alfonso et al., 2019). As with CypA, in the present study, CypC was also elevated in patients with CAD. Lately, our group has proposed CypC levels >17.5 pg/mL as a new biomarker of CAD and has described its correlation with cardiovascular risk factors (Alfonso et al., 2019; Bayon et al., 2020; Bayon et al., 2021). Nevertheless, this is the first time that the association of CypC with high glucose and total cholesterol serum levels has been described in CAD patients. Although little is known about the extracellular functions of CypC, it has been recurrently related to inflammation and CVD. This protein participates in the endoplasmic reticulum (ER) homeostasis and is involved in B and T-cell differentiation and macrophage activation (Paiva et al., 2021). Furthermore, increased CypC levels were found after focal cerebral ischemia (Shimizu et al., 2005). Therefore, the present data strengthen the relationship of CypC with inflammation and CVD. Moreover, both CypA and C were associated with HTA, DL, and tobacco in CAD patients with a robust OR when other cardiovascular risk factors were also included, reinforcing the predictive value of these proteins in CAD.

CypB participates in physiological processes, such as calcium and ER homeostasis, collagen folding, and prolactin signaling (Zhao et al., 2017). Nevertheless, CypB has been related to cancer, neuroinflammation, and CVD, among others. Also, this immunophilin participates in the pathogenesis of atherosclerosis and HTA by promoting vascular smooth muscle cell growth and ROS generation (Perrucci et al., 2015). In the present study, CypB serum levels were higher in females than in male controls. This sex-difference in serum Cyps levels could be due to distinct hormone and gene expression in women (Fang et al., 2009). Sex-based differences are also noted in the atherosclerosis scenario. In this sense, estrogen has a cardioprotective effect in women, however, these protective effects disappeared after menopause (Vakhtangadze et al., 2021). Therefore, CypB cannot be used as a biomarker in women. To avoid interferences, only the male population was used in the studies of CypB. In this population, CypB was elevated in patients with CAD, associating this protein with CAD for the first time. Moreover, CypB serum levels were significantly elevated in patients with CAD and T2D or HTA, being the first time to be related to diabetes. Furthermore, multivariate logistic regression analysis demonstrates that CypB >63.26 pg/mL is a risk factor for the presence of CAD along with HTA, DL, and high glucose serum levels in males. Moreover, there is a correlation between CypB serum levels and T2D. Thus, high serum levels of CypB in patients with CAD might provide prognostic information on T2D status. Moreover, CypB was also implicated in other metabolic or hormone-mediated processes. In this regard, it has been demonstrated its relationship with the atrial natriuretic peptide, prolactin, and metabolic syndrome, among others (Rycyzyn et al., 2000; Zhang et al., 2021). Therefore, the high CypB levels could have a metabolic and/or hormone course. On the other hand, CypB has been also correlated with glucose levels above 100 mg/dL. Hyperglycaemia can contribute to microvascular dysfunction and MI, increasing the risk of rehospitalization for chest pain, regardless of diabetic status (Mone et al., 2021; Mone et al., 2023). Furthermore, the management and control of glucose levels are beneficial in reducing long-term mortality after acute myocardial infarction (Cui et al., 2022).

The immunophilin CypD also plays an important function in atherosclerosis and CAD. This protein is the main regulator of mitochondrial pore opening, which upon inflammation or oxidative stress leads to mitochondrial dysfunction (Amanakis and MurphyCyclophilin, 2020). The mitochondrial pore and thus CypD, are implicated in IR injury in the heart, brain, and kidney. Therefore, this Cyp has a key role in chronic inflammation and numerous cardiovascular diseases (Perrucci et al., 2015). However, in the present study, no differences were found in serum CypD levels of patients with CAD. These data are in line with the previous data of our group (Alfonso et al., 2019; Bayon et al., 2020). This may be indicating that the role of CypD in CAD is mainly intracellular rather than extracellular. Non-etheless, further studies should be done to better understand the extracellular role of this protein and its relevance in CVD.

Former screening and therapeutic guidelines focus on cholesterol as the primary biomarker of CVD. Normally, LDLc levels above 100 mg/dL and HDLc levels below 40 mg/dL are considered cardiovascular risk factors (Vekic et al., 2022). However, in this study, LDLc levels were lower in CAD patients than in controls, due to statin treatment. Therefore, Cyps have a better prognostic for CAD presence in this study population. Additionally, Cyps levels could be used to assess the therapeutic effects of medical treatments. In this sense, treatment to control atherosclerosis risk factors also decreased CypA levels in patients with CAD (Satoh et al., 2013).

In conclusion, the present study validates the prognostic value of CypA and CypC in CAD and reinforces their relationship with cardiovascular risk factors. Furthermore, it demonstrates for the first time that CypB levels above 63.26 pg/mL have the potential as a biomarker for CAD when male patients suffer from DL or HTA. Moreover, CypB is correlated with T2D and other cardiovascular risk factors. In this sense, Cyp levels could be used for the diagnosis of CAD disease. Moreover, CypA, B, and C could serve as potential novel therapeutic strategies for CAD.

## Study limitations

Some limitations should be addressed. The study population was relatively small for T2D patients. Nevertheless, in this small population, the results were statistically significant. In this sense, this limitation did not influence the reliability of the results of the present study. Moreover, women with diabetes are more likely to have cardiovascular disease than men. In this sense, further analysis in a larger population, including more women, will clarify the importance of Cyps in CAD. In addition, there was an effect of the consumption of lipid-lowering agents on LDLc levels, since LDLc levels were lower in patients with CAD than in the control group.

## Data Availability

The raw data supporting the conclusions of this article will be made available by the authors, without undue reservation.

## Abbreviations

CAD: coronary artery disease; CI: confidence interval; CVD: cardiovascular disease; Cyp: cyclophilin; DL: dyslipidemia; ER: endoplasmic reticulum; HDLc: high-density lipoprotein cholesterol; Hs-CRP: high-sensitivity C-reactive; HTA: hypertension; IR: ischemia-reperfusion; LDL: low-density lipoprotein; LDLc: low-density lipoprotein cholesterol; MI: myocardial infarction; mPTP: mitochondrial permeability transition pore; NPV: negative predictive value; OR: odds ratio; PC: principal component; PPIase: peptidyl-prolyl cis/trans isomerase; PPV: positive predictive value; ROC: receiver-operating-characteristic curves; T2D: type 2 diabetes; TG: triglycerides.
